# Supplementary figures and images for: Perioperative management of kidney transplantation in China: A national survey in 2021
Source: PLoS One. 2024 Feb 14;19(2):e0298051. doi: 10.1371/journal.pone.0298051 (PMC10866523; doi:10.1371/journal.pone.0298051)

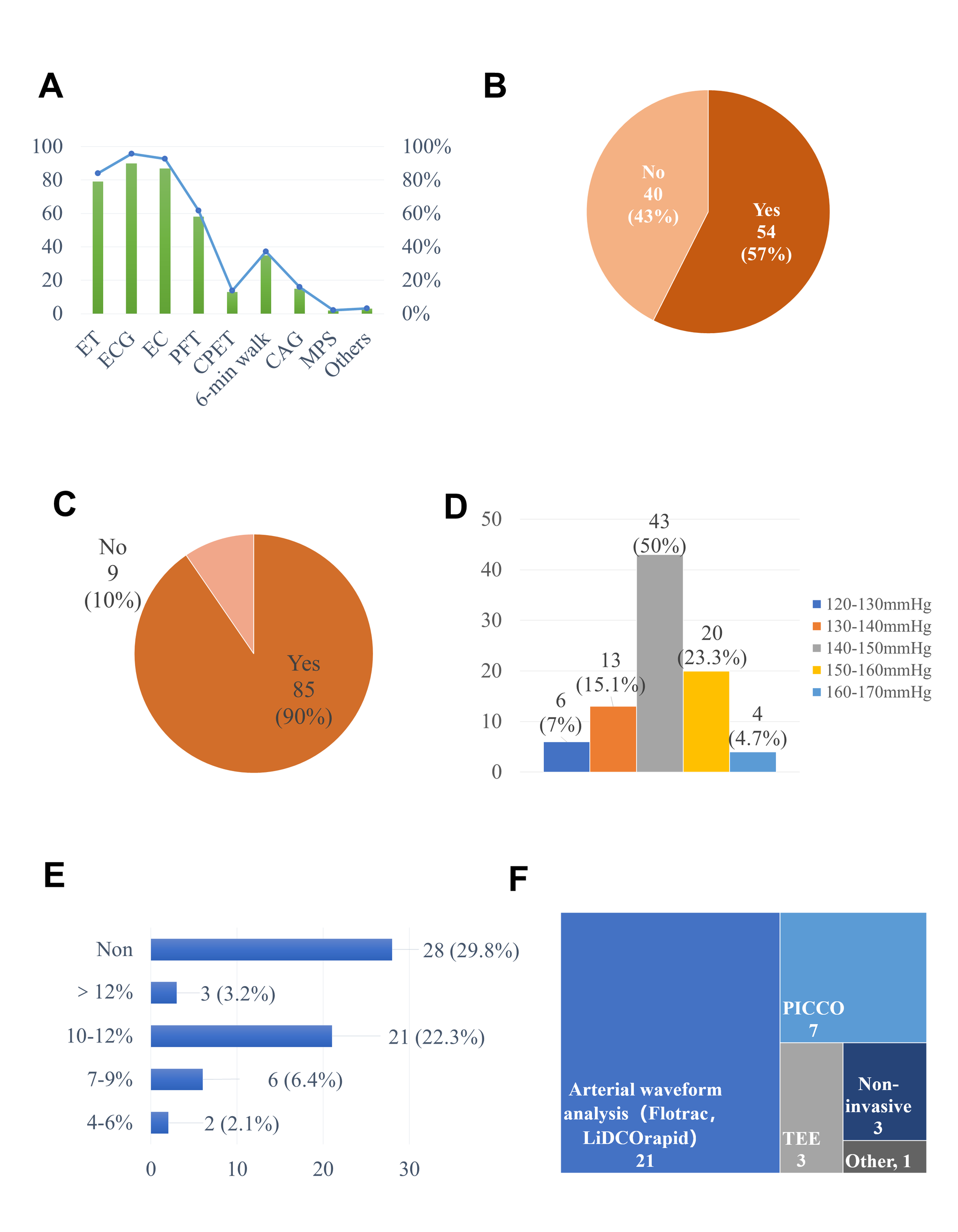

Supplement: S1 Fig — A, Preoperative cardiopulmonary function assessments. The column represents the number of hospitals, and the dot represents the percentage. B, Status of routine use of albumin. C, Investigation on the routinely use of invasive arterial monitor. D, The specific targeted value of intraoperative systolic blood pressure (mmHg) in renal transplant recipients after renal artery opening. E, Specific target of CVP range (cmH2O) after renal artery opening. F, CO monitoring methods employed during renal transplantation. ET, Exercise tolerance; ECG, electrocardiogram; EC, Echocardiography; PFT, Pulmonary function tests; CPET, cardiopulmonary exercise test; CAG, coronary angiogram; MPS, Myocardial perfusion scan; SBP, systolic blood pressure; CVP, central venous pressure; CO, cardiac output; PICCO, Pulse indicator Continuous Cardiac Output; TEE, transesophageal echocardiography. (TIF) [file pone.0298051.s001.tif]

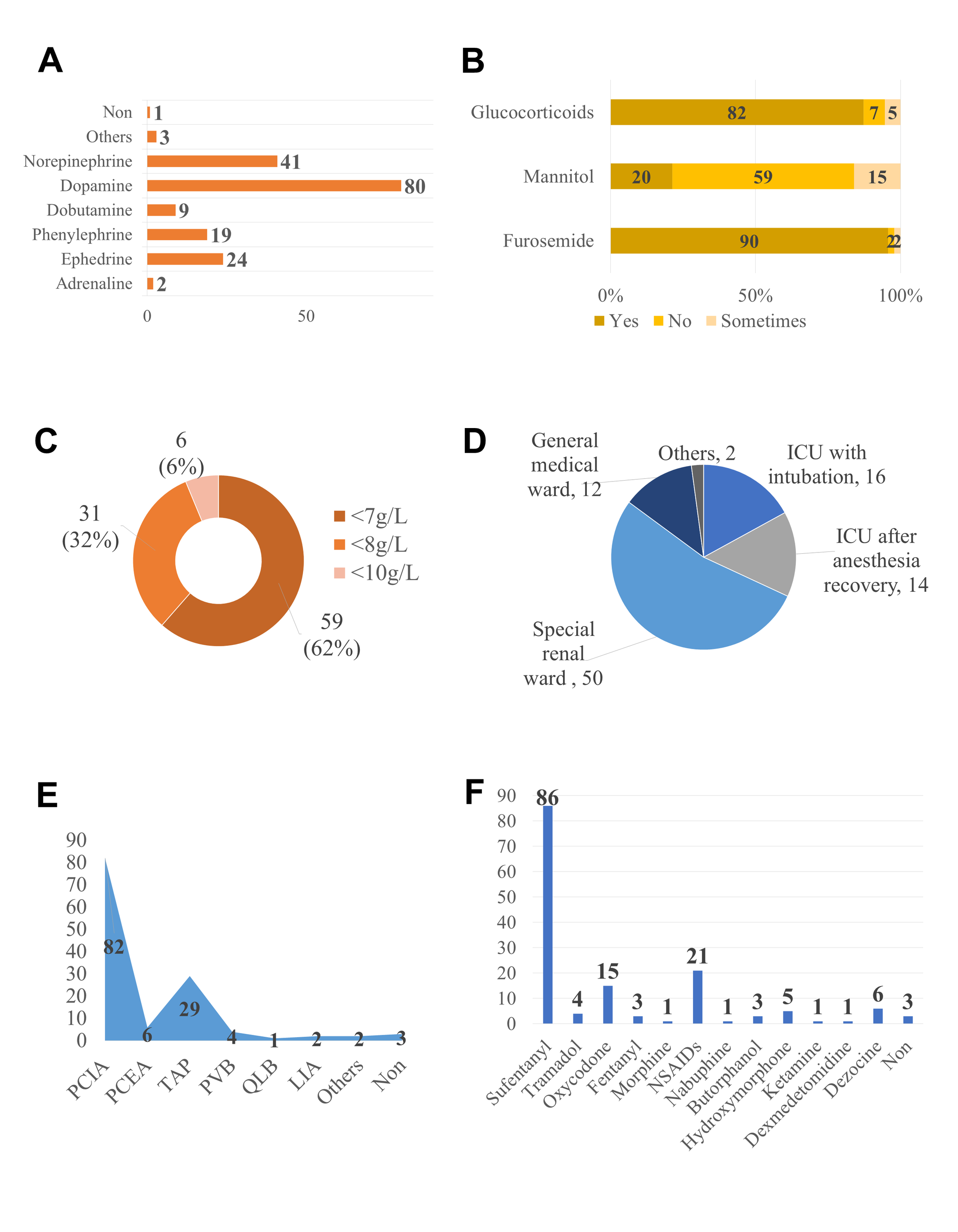

Supplement: S2 Fig — A, Most commonly used vasoactive drugs. B, The routinely use of furosemide, Mannitol and Glucocorticoids. C, The limit value of Hemoglobin for starting the transfusion. D, The postoperative destination of renal transplant recipients. E, The main methods of postoperative analgesia after kidney transplantation. F, The most commonly used intravenous analgesics in PCIA. PCIA, patient-controlled intravenous analgesia; PCEA, patient controlled epidural analgesia; TAP, Transversus Abdominis Plane block; PVB, paravertebral block; QLB, lumbar quadratus muscle block; LIA, local infiltration anesthesia; ICU, intensive care unit. (TIF) [file pone.0298051.s002.tif]
