# Supplementary material for: Perioperative management of kidney transplantation in China: A national survey in 2021
Source: PLoS One. 2024 Feb 14;19(2):e0298051. doi: 10.1371/journal.pone.0298051 (PMC10866523; doi:10.1371/journal.pone.0298051)
Supplement: S1 File — (DOCX) [file pone.0298051.s003.docx]

**A national survey for perioperative anaesthetic management of kidney transplantation**

Dear Sir/Madam:

Kidney transplantation is the preferred treatment for end-stage renal disease (ESRD) developed from advanced and irreversible CKD. In 2019, the number of CKD patients reached 150.5 million in China, accounting for 10.6% of the total population. Despite many progresses in renal transplant surgery, the risk of perioperative complications remains. With the growth use of marginal organs for renal transplantation and aging of donors as well as recipients, optimized perioperative management to improve the renal outcome has become a new challenge, in which the role of anaesthesiologists is assignable. Although various guidelines for kidney transplantation have been published globally, there is little national consensus on anaesthesia management for kidney transplantation and the actual clinical practice according to guidelines remain unknown in China. Thus, we developed a survey to generate an overview of current situation of national practice across Chinese renal transplant units to identify the heterogeneity and provide evidence for improving perioperative management of kidney transplantation.

Before answering this questionnaire, please be aware of the following content: There are 44 questions in the questionnaire and will take approximately 30 min to complete. This survey will collect the name, sex, age and phone number of the participants to validate the data filled. Before filling the questionnaire, please prepare the following data: number of kidney transplants in 2021 and the clinical practice of anaesthesia management occurred in your center. The privacy of the data would be stored and maintained in password-protected computers and any identifying information would be delinked after analysis and publication. The questions can be answered only after you agree with these items. Once you agree to answer this questionnaire with your name filled in, it will be considered as obtaining your informed consent. Please make sure that the choice you select and the numbers you fill in are accurate and reliable.

Thank you very much for your contribution to China’s comfortable medical care!

Yours, sincerely

Diansan Su

May, 2022

1. Please fill in your personal information so that we can check the questionnaires later. Thank you!

Name _________

Sex □male □female

age _____years

phone number ________________

1. Please fill in full name of your affiliation.
2. What is your department？

□department of anaesthesiology □other，please specify：__________

1. What is your current responsibility？

□director in charge of kidney transplantation anaesthesia □other，please specify：__________

1. In which year did your transplant unit start kidney transplantation？
2. Number of kidney transplants in 2021？ （please specify the exact number）

□0-50 □50-100 □100-200 □200-300 □300-400 □>400

1. What kind of kidney transplantation does your center currently focus on？

□Donor kidney transplantation □Cadaveric kidney transplantation

1. Does your center have a specific guideline for the perioperative management of kidney transplantation?

□Yes □No

1. Whether prophylactic antibiotics are used before operation (before incision)？

□Yes □No

1. Whether hypertension is regularly controlled before operation？

□Yes □No

1. Whether glycemia is regularly controlled before operation？

□Yes □No

1. Which tests do you routinely request to assess preoperative cardiopulmonary function in patients to be listed for renal transplantation (Multiple Choice Questions, MCQ)？

□Exercise tolerance □6-min walk □electrocardiogram □Transthoracic echocardiography (TTE) □pulmonary function tests □myocardial perfusion scan

□cardiopulmonary exercise test □coronary angiogram □Others，please specify：______________

□cardiopulmonary function tests are not routinely assessed

1. What is the main anaesthesia method selected during renal transplantation？

□general anaesthesia(GA) □regional block anaesthesia（including nerve block, spinal anaesthesia, epidural anaesthesia, etc）

□GA combined with regional block anaesthesia □Others，please specify：_________

1. What is the main anaesthesia scheme used for general anaesthesia during renal transplantation?

□Total Intravenous Anaesthesia(TIVA) □intravenous-inhalation combined general anaesthesia □inhaled anaesthesia

1. During the induction period of general anaesthesia for kidney transplantation，what are the main intravenous anaesthetics used in your center（MCQ）？

□Propofol □Etomidate □Midazolam □Sufentanyl □Fentanyl

□Succinylcholine □Rocuronium □Atracurium □Cisatracurium

□Others，please specify：_____________

1. During the maintenance of anaesthesia, which inhaled anaesthetic is most commonly selected in your center for kidney transplantation?

□Sevoflurane □Isoflurane □Desflurane □Nitrous oxide □Not use inhalation anaesthetics

□Others，please specify：_____________

1. During the maintenance of anaesthesia, which intravenous anaesthetics are most commonly used in your center for kidney transplantation(MCQ)？

□Propofol □Sufentanyl □Fentanyl

□Depolarizing Muscle Relaxants □Non-depolarizing Muscle Relaxants □Others，please specify：_____________

1. Whether the depth of anaesthesia is routinely monitored during operation？

□Yes □No

1. What is your main intraoperative fluid regime during renal transplantation？

□Goal-directed hemodynamic therapy(GDFT) □CVP-guided □experience-guided □Others，please specify: ______________________

1. If you answered GDFT to Question 23, according to which goal orientation do you guide intraoperative fluid management？

□SVV(Stroke volume variation) □PPV(Pulse pressure variation) □Others，please specify: __________________________

1. Whether artificial colloid is routinely infused during kidney transplantation？

□Yes □No

1. If you answered YES to Question 21, which artificial colloid is predominantly used intraoperatively in your center？

□Dextran □Gelatin □Hydroxyethyl starch □Other，please specify: ___________________

1. Whether albumin is routinely infused during kidney transplantation in your center？

□Yes □No □If yes，what is total dose？_____g

1. Which crystalloids is predominantly used intraoperatively in your center？

□Normal saline □Lactic acid sodium solution □Glucose solution □Other，please specify: ________________

1. Do you regularly monitor CVP for kidney transplantation by central venous catheters (CVCs)？

□Yes □No □Not clear

1. If you answered YES to Question 25, what is the common route of CVCs？

□Internal jugular vein □Subclavian vein □Supraclavicular vein □Other，please specify:__________________

1. If you answered yes to Question 25, do you target a specific CVP value or range after renal artery opening？

□Yes □No

1. If you answered yes to Question 27, what is the specific target of CVP range（cmH2O）after renal artery opening？

□<4% □4-6% □7-9% □10-12% □>12%

1. What do you anticipate the average operative blood loss to be during renal transplant?？

□<100ml □100-250ml □250-500ml □500-750ml □>750ml

1. Do you routinely insert arterial lines into patients undergoing renal transplantation？

□Yes □No

1. Do you target specific intraoperative SBP (systolic blood pressure) values（mmHg） in renal transplant recipients after renal artery opening？

□Yes □No

1. If you answered yes to Question31，what is the specific SBP range targeted（mmHg）？

□<120 □120-130 □130-140 □140-150 □150-160 □160-170 □170-180 □>180

1. Does your center regularly use cardiac output (CO) monitoring during renal transplantation？

□Yes □No □Sometimes

1. If your center does regularly use CO monitoring, which methods are employed (MCQ)？

□TEE (Transesophageal echocardiography) □Arterial waveform analysis（Flotrac，LiDCOrapid） □Swan-Ganz catheter □PICCO (Pulse indicator Continuous Cardiac Output)

□Non-invasive □Bioelectrical impedance technique

□Others，please specify:__________________

1. Do you regularly perform TEE or TTE during renal transplantation?

□Yes □No

1. Which vasoactive drugs does your center most commonly use（MCQ）？

□Dopamine □Ephedrine □Dobutamine □Phenylephrine □Norepinephrine

□Adrenaline □Other，please specify:_____________

1. Are the following drugs routinely used during kidney transplantation? Furosemide？Mannitol？ Glucocorticoids？

□Yes □No □Sometimes

1. If blood transfusion is required during kidney transplantation, what is the limit value of Hb for starting the transfusion in your center?

□<7g/L □<8g/L □<10g/L

1. Do you routinely monitor intraoperative temperature during kidney transplantation？

□Yes □No □Sometimes

1. Are measures of maintaining temperature regularly applied for kidney transplantation in your center？

□Yes □No □Sometimes

1. Regarding postoperative analgesia, what are the main methods to relieve pain after kidney transplantation（MCQ）？

□PCA（patient-controlled analgesia） □PCEA（patient controlled epidural analgesia） □TAP(Transversus Abdominis Plane block) □paravertebral block □Other，please specify：__________

1. If PCA is used, the most commonly used intravenous analgesics in PCA for post-renal transplantation is（MCQ）？

□Fentanyl □Sufentanyl □Morphine □Oxycodone □Tramadol □NSAIDs (non-steroidal anti-inflammatory drug)

□Other，please specify：______________

1. Do you regularly use antagonism for nondepolarizing muscle relaxants on kidney transplantation？

□No □Yes (Antagonism by routine use of neostigmine and atropine)

□Yes (Sugammadex is routinely used when rocuronium is applied)

□Yes (Sugammadex is occasionally used when rocuronium is applied)

1. What is the postoperative destination of renal transplant recipients in your center？

□ICU(Intensive care unit) with intubation □ICU after anaesthesia recovery □Special renal ward □General medical ward

□Other，please specify：___________
