# Supplementary material for: Perioperative management of kidney transplantation in China: A national survey in 2021
Source: PLoS One. 2024 Feb 14;19(2):e0298051. doi: 10.1371/journal.pone.0298051 (PMC10866523; doi:10.1371/journal.pone.0298051)
Supplement: S1 Table — KT, Kidney transplantation. (DOCX) [file pone.0298051.s004.docx]

**S1 Table. Information of replied centers and official centers divided by province in 2021**

|  | **Replied centers** | **official centers** | **KT cases of replied centers** | **KT cases of official centers** | **Response rate** | **Rate of KT cases by replied centers** |
| --- | --- | --- | --- | --- | --- | --- |
| Anhui | 3 | 3 | 522 | 522 | 100.0% | 100.0% |
| Beijing | 9 | 11 | 715 | 715 | 81.8% | 100.0% |
| Fujian | 2 | 2 | 20 | 20 | 100.0% | 100.0% |
| Gansu | 2 | 2 | 18 | 18 | 100.0% | 100.0% |
| Guangdong | 11 | 16 | 1085 | 1352 | 68.8% | 80.3% |
| Guangxi | 3 | 3 | 603 | 603 | 100.0% | 100.0% |
| Guizhou | 3 | 3 | 263 | 263 | 100.0% | 100.0% |
| Hainan | 2 | 2 | 139 | 139 | 100.0% | 100.0% |
| Hebei | 1 | 1 | 53 | 53 | 100.0% | 100.0% |
| Henan | 4 | 5 | 856 | 873 | 80.0% | 98.1% |
| Heilongjiang | 1 | 2 | 9 | 21 | 50.0% | 42.9% |
| Hubei | 4 | 5 | 737 | 779 | 80.0% | 94.6% |
| Hunan | 7 | 10 | 775 | 827 | 70.0% | 93.7% |
| Jilin | 1 | 1 | 351 | 351 | 100.0% | 100.0% |
| Jiangsu | 4 | 5 | 174 | 187 | 80.0% | 93.0% |
| Jiangxi | 3 | 3 | 362 | 362 | 100.0% | 100.0% |
| Liaoning | 3 | 4 | 132 | 136 | 75.0% | 97.1% |
| Inner Mangoria | 1 | 1 | 19 | 19 | 100.0% | 100.0% |
| Ningxia | 1 | 1 | 0 | 0 | 100.0% | 0 |
| Qinghai | 1 | 1 | 0 | 0 | 100.0% | 0 |
| Shandong | 7 | 7 | 631 | 631 | 100.0% | 100.0% |
| Shanxi | 2 | 3 | 248 | 259 | 66.7% | 95.8% |
| Shaanxi | 1 | 2 | 392 | 396 | 50.0% | 99.0% |
| Shanghai | 4 | 5 | 501 | 515 | 80.0% | 97.3% |
| Sichuan | 2 | 2 | 734 | 734 | 100.0% | 100.0% |
| Tianjing | 1 | 1 | 148 | 148 | 100.0% | 100.0% |
| Tibet | 0 | 1 | 0 | 0 | 0 | 0 |
| Xinjiang | 1 | 2 | 7 | 14 | 50.0% | 50.0% |
| Yunnan | 2 | 2 | 145 | 145 | 100.0% | 100.0% |
| Zhejiang | 6 | 7 | 694 | 873 | 85.7% | 79.5% |
| Chongqing | 2 | 2 | 71 | 71 | 100.0% | 100.0% |
